# Fires Due to Air Raids

**Published:** 1915-05-22

**Authors:** Ellis Marsland

**Affiliations:** Gen. Hon. Secretary. The British Fire Prevention Committee


### THE EDITOR'S LETTER-BOX.

### Fires Due to Air Raids.

*To the Editor of* The Hospital.

Sir,—As it is within the bounds of possibility that attempts may be made to start incendiary fires, the British Fire Prevention Committee make the following suggestions: (*a*) The various fire brigades cannot be expected successfully to deal with any large number of outbreaks, and their attention would in any case be primarily devoted to fires affecting Government offices, workshops engaged on Government work, military stores, docks and hospitals, and the like, rather than to private property or semi-public property, however valuable. It therefore behoves all owners and occupiers of such premises to do their best to organise for self-help.

(*b*) Innumerable establishments, such as factories, warehouses, stores, modern office blocks, flats, hotels, and large private houses, as also the semi-public establishments like schools, baths, theatres, music-halls, and assembly rooms, are equipped with internal hydrant services and first-aid fire appliances. These should be promptly examined, and the staffs (both male and female) frequently practised in handling the gear. The smaller establishments, such as shops, offices, and ordinary domestic buildings, should have an ample supply of buckets (kept ready filled with water) and, where funds permit, one or more small hand-pumps. Occupants should know how to use these appliances efficiently.

(*c*) Every effort should be made by the public to assist the fire brigades in their onerous duties, but they should not omit on any account immediately to notify any outbreak that comes to their notice by telephone or otherwise—the method of calling assistance to be posted up in a conspicuous position.

In any event, the British Fire Prevention Committee consider the public would be well advised to have these matters attended to in the course of the present month, and should remember that fires caused by incendiary bombs, if *promptly* tackled with water in fair bulk, force, and continuity, need not spread, and are not quite the terrifying problem to deal with that the enemy would make them.

Those who desire additional useful hints on the fire question arising from possible air raids should apply for the British Fire Prevention Committee's "Warnings," which are provided gratuitously upon written application to the Registrar at 8 Waterloo Place, Pall Mall, London, S.W. (enclosing large size stamped and addressed envelope). These "Warnings" give useful information as to the necessary precautions and how to act when the emergency arises. Where special technical advice is desired application should be made in writing, which will either be dealt with direct free, or, where it is a matter affecting local authorities, forwarded in the proper direction for attention.—Yours very truly,

Ellis Marsland, Gen. Hon. Secretary.

The British Fire Prevention Committee,
May 17, 1915.

